# Chromosome-Biased Binding and Gene Regulation by the *Caenorhabditis elegans* DRM Complex

**DOI:** 10.1371/journal.pgen.1002074

**Published:** 2011-05-12

**Authors:** Tomoko M. Tabuchi, Bart Deplancke, Naoki Osato, Lihua J. Zhu, M. Inmaculada Barrasa, Melissa M. Harrison, H. Robert Horvitz, Albertha J. M. Walhout, Kirsten A. Hagstrom

**Affiliations:** 1 Program in Molecular Medicine and Program in Cell Dynamics, University of Massachusetts Medical School, Worcester, Massachusetts, United States of America; 2 Program in Gene Function and Expression and Program in Molecular Medicine, University of Massachusetts Medical School, Worcester, Massachusetts, United States of America; 3 Howard Hughes Medical Institute, Department of Biology, Massachusetts Institute of Technology, Cambridge, Massachusetts, United States of America; Massachusetts General Hospital, Howard Hughes Medical Institute, United States of America

## Abstract

DRM is a conserved transcription factor complex that includes E2F/DP and pRB family proteins and plays important roles in development and cancer. Here we describe new aspects of DRM binding and function revealed through genome-wide analyses of the *Caenorhabditis elegans* DRM subunit LIN-54. We show that LIN-54 DNA-binding activity recruits DRM to promoters enriched for adjacent putative E2F/DP and LIN-54 binding sites, suggesting that these two DNA–binding moieties together direct DRM to its target genes. Chromatin immunoprecipitation and gene expression profiling reveals conserved roles for DRM in regulating genes involved in cell division, development, and reproduction. We find that LIN-54 promotes expression of reproduction genes in the germline, but prevents ectopic activation of germline-specific genes in embryonic soma. Strikingly, *C. elegans* DRM does not act uniformly throughout the genome: the DRM recruitment motif, DRM binding, and DRM-regulated embryonic genes are all under-represented on the X chromosome. However, germline genes down-regulated in *lin-54* mutants are over-represented on the X chromosome. We discuss models for how loss of autosome-bound DRM may enhance germline X chromosome silencing. We propose that autosome-enriched binding of DRM arose in *C. elegans* as a consequence of germline X chromosome silencing and the evolutionary redistribution of germline-expressed and essential target genes to autosomes. Sex chromosome gene regulation may thus have profound evolutionary effects on genome organization and transcriptional regulatory networks.

## Author Summary

X chromosomes differ in number between the sexes and differ from autosomes in their associated proteins and gene regulatory properties. In *C. elegans* both X chromosomes are partially silenced in hermaphrodite germlines. Germline-expressed and essential genes are autosome-enriched and are thought to have fled the X chromosome during evolution because silencing these genes would result in sterility or lethality. We discovered that the *C. elegans* DRM complex, which controls transcription of genes implicated in development and cancer, avoids the X chromosome. We first describe how DNA–binding components of the DRM complex together recognize DNA sequences upstream of its target genes, and we describe that DRM controls different target genes in the germline versus the soma. We show that the DRM binding motif, the genes bound by DRM, and the embryonic genes regulated by DRM are all under-represented on the X chromosome. Interestingly, compromising DRM function in the germline enhances X chromosome silencing, and we discuss how autosome-bound DRM might regulate X-linked genes in *trans*. We propose that autosome-enriched binding of DRM co-evolved with the redistribution of its germline-expressed and essential target genes to autosomes. Our data highlight how X chromosome gene regulation may impact both the genomic distribution of gene sets and their transcriptional regulators.

## Introduction

The development of multi-cellular organisms is orchestrated by transcription factors that coordinate the spatiotemporal expression of sets of target genes. Transcription factors often act together in the context of multi-protein complexes. For instance, DREAM is a multi-protein complex conserved among *Caenorhabditis elegans* (DRM), *Drosophila melanogaster* (dREAM/Myb-MuvB) and *Homo sapiens* (hDREAM or LINC), and includes a retinoblastoma tumor suppressor pRb-family protein and the DNA binding heterodimer E2F/DP [1]–[7]. DREAM coordinates the expression of cell division and differentiation genes during development, and its subunit activities are altered in many human tumors [8].

In *C. elegans*, the genes that encode DRM subunits were originally identified in genetic screens for mutations causing defects in vulva development. Specifically, DRM subunits are encoded by synMuvB (synthetic multivulva class B) genes, which act “synthetically” with synMuvA genes to antagonize Ras signaling during vulva development [9]–[12]. Most synMuvB genes are broadly expressed chromatin-associated transcriptional regulators, and when mutated affect a range of biological processes including embryo polarity [13], apoptosis [14], [15], sex determination [16], and RNA interference [17], [18]. Despite their important roles in disparate developmental contexts, a genome-wide analysis of genes bound and regulated by synMuvB proteins is lacking.

Biochemical studies of *D. melanogaster* identified the dREAM/Myb-Muv-B complex and a partially overlapping testes-specific complex called tMAC [1]–[3], [19], [20]. These complexes contain homologs of *C. elegans* synMuvB proteins. dREAM-like protein complexes were subsequently identified from *C. elegans* (DRM, [4]) and human cells (hDREAM/LINC, [5], [7]). DRM includes LIN-35(Rb), EFL-1(E2F), DPL-1(DP), LIN-54(Mip120), LIN-9(Mip130), LIN-37, LIN-52, and LIN-53(Caf1). The human and fly complexes share these subunits and additionally contain a Myb subunit that is not apparent in *C. elegans* (Figure S1A).

Several DREAM subunits contribute to its sequence-specific DNA binding, including E2F and DP, which together bind DNA as a heterodimer, and Myb. In flies and humans, E2F/DP and Myb act in a mutually exclusive manner to direct DREAM to its target genes [5]–[7], [21]. Human DREAM is targeted to different sets of promoters by subunit switching [5]–[7]. During the G0 phase of the cell cycle, the DREAM complex incorporates the Rb-family protein p130 and E2F4, but not Myb, to repress S phase genes. At cell cycle entry, p130 and E2F4 dissociate from the complex, and Myb is incorporated to promote activation of M phase genes. LIN-54 is another DREAM component that has been reported to bind DNA: *D. melanogaster* Mip120(Lin54) binds specific sequence elements within the chorion gene cluster [1], *C. elegans* LIN-54 binds promoters in yeast one-hybrid (Y1H) assays [22], and human Lin54 interacts with the human *cdc2* promoter *in vitro* [23]. However, the overall contribution of LIN-54 DNA binding to DREAM complex function has not yet been explored.

Genome-wide binding and expression profiling studies of DREAM in mammalian cell culture primarily identified cell cycle genes as targets for the complex [5], while *D. melanogaster* cultured cell studies additionally revealed targets with sex- and development-specific expression [2], [21], [24]. Thus, it is not clear whether developmental gene regulation is a conserved DRM function. With the exception of gene expression profiling of the *C. elegans* germline [25], genome-scale studies of the DREAM complex were performed in cultured differentiated cells. It is important to extend genome-wide analyses of DREAM to multiple cell types and tissues derived from intact organisms, to enable assessment of DREAM function through development.

A key developmental function of *D. melanogaster* and *C. elegans* DRM subunits is the regulation of gene expression in the germline [19], [20], [25], which must occur within the context of specialized germline gene expression features. The first such feature is a germline-specific form of X chromosome silencing. In male germlines of many species the single X is transcriptionally inactive and in *C. elegans* hermaphrodite germlines the two X chromosomes are partially silenced [26], [27]. Whether transcription factors like DREAM act equally on X-linked and autosomal genes, which exist in different chromatin regulatory environments, is not known. The second property special to germline-expressed genes is that they primarily reside on autosomes, possibly because of an evolutionary adaptation to X silencing [28]–[31]. It has not been explored whether the chromosome-biased location of germline differentiation genes is related to chromosome-biased binding sites and chromosome-biased regulation by distinct transcription regulatory networks.

Here we analyze genome-wide binding and function of *C. elegans* LIN-54. We demonstrate that LIN-54 DNA-binding activity is required for the DRM complex to efficiently bind and regulate target genes containing adjacent putative E2F/DP and LIN-54 binding sites. We show that LIN-54 binds to the promoters of genes involved in cell division, development, and reproduction, and acts differently in the germline versus the soma. The E2F/DP-LIN-54 binding motif, individual target genes, and overall DRM function are conserved among worms, flies, and humans. Despite this conservation, we discovered one striking feature of *C. elegans* DRM not shared in flies or humans: it is depleted from X chromosomes. We show that DRM binding, the E2F-LIN-54 hybrid motif, and LIN-54-regulated genes are all autosome-enriched. One paradoxical exception occurs in the germline, where DRM binds autosomes but genes down-regulated in DRM mutants are enriched on X chromosomes. Evolutionary pressures imposed by germline X chromosome silencing in *C. elegans* are thought to have resulted in the autosome-biased location of germline-expressed and essential genes, major targets of DRM-mediated regulation. We propose that the autosome bias of *C. elegans* DRM co-evolved with the redistribution of its target genes. This example illustrates how sex chromosome gene regulation may create a biased genomic location of gene sets and their transcriptional regulatory networks.

## Results

### LIN-54 Binds DNA through Its Tesmin Domains

**Figure 1:**
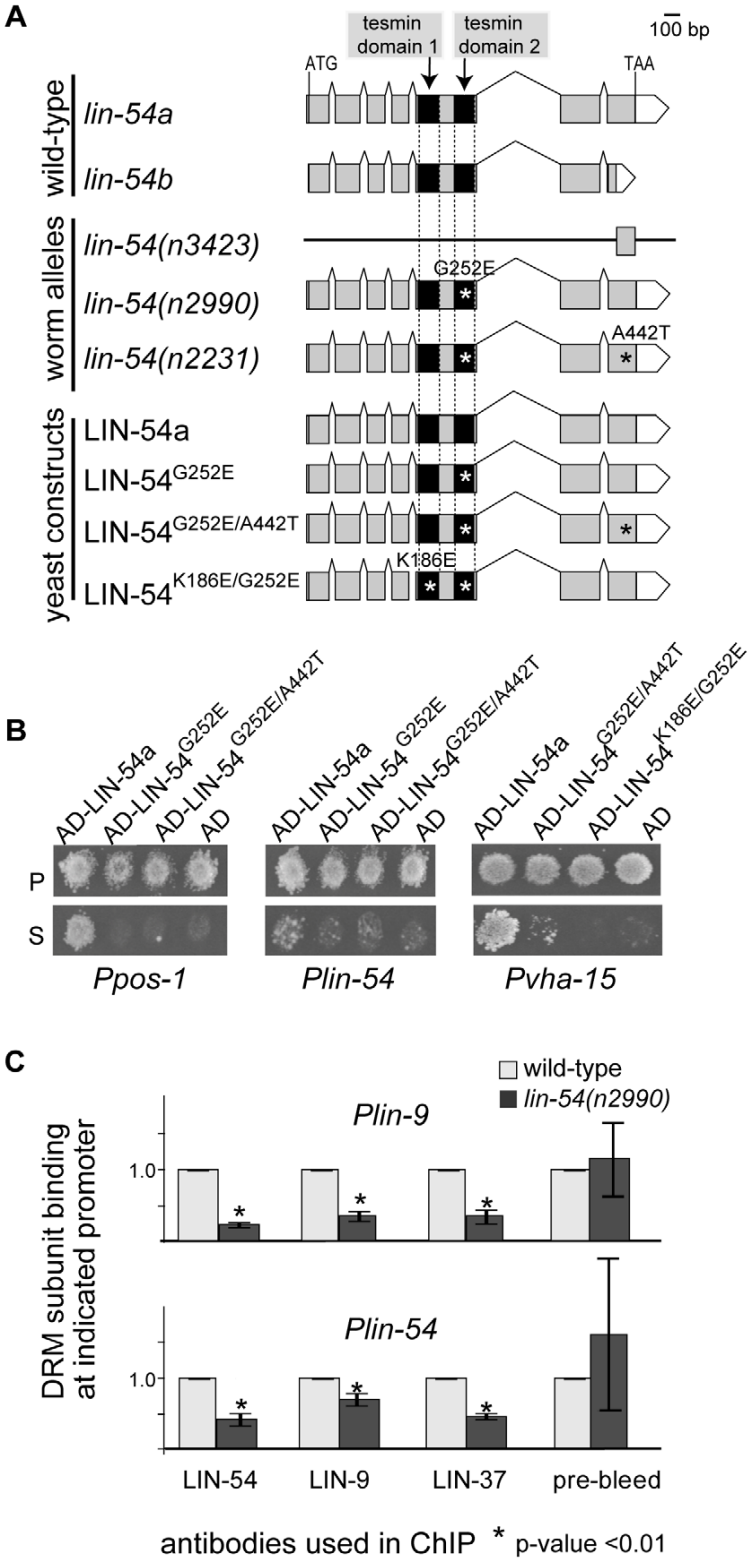
LIN-54 binds DNA directly through its tesmin domains and recruits DRM to promoters. *(A) C. elegans lin-54* gene structure for wild-type isoforms *(lin-54a* and *lin-54b)*, *lin-54* mutant alleles, and yeast constructs used in this study. The *lin-54* gene encodes a protein with two tesmin/CXC domains (black boxes). *lin-54(n3423)* is a null allele in which the 5′ end and most exons are deleted. *lin-54(n2990)* is a missense allele that harbors a mutation in the second tesmin domain, and *lin-54(n2231)* has both the tesmin domain mutation and an additional point mutation. Constructs equivalent to *lin-54a*, *lin-54(n2990)*, and *lin-54(n2231)* were used in yeast one-hybrid (Y1H) assays, and are referred to as LIN-54a, LIN-54**^G252E^**, and LIN-54**^G252E/A442T^**, respectively. An additional LIN-54 construct containing a point mutation in each tesmin domain was created and is referred to as LIN-54**^K186E/G252E^**. Gray box = exon, black box = tesmin domain, white box = 3′ untranslated region, asterisk = missense mutation. *(B)* Y1H assays using wild-type LIN-54a, LIN-54**^G252E^**, LIN-54**^G252E&A442T^**, and LIN-54**^K186E/G252E^** mutant proteins with the promoters of the genes *pos-1*, *lin-54*, and *vha-15*. AD = Gal4 activation domain, P = permissive media, S = selective media. *(C)* DRM subunit binding in wild-type and *lin-54(n2990)* mutants, measured by ChIP-qPCR at the target promoters *lin-9* and *lin-54*. Binding is shown as the amount of DNA amplified in each ChIP sample relative to input, with the ratio in wild-type set to 1.0. Standard deviations from three independent experiments are shown.

The *lin-54* gene encodes two proteins, LIN-54a and LIN-54b, both of which contain two tandem cysteine-rich repeats known as the tesmin/CXC domain (Figure 1A). Genetic screens for synMuv vulva development phenotypes identified the *lin-54(n2990)* and *lin-54(n2231)* missense alleles which confer similar loss-of-function phenotypes as a *lin-54(n3423)* deletion mutant [4], [11]. These missense alleles were independently isolated and contain the same single-base substitution in the second tesmin domain (tesmin domain 2), which changes glycine 252 to a glutamic acid (G252E). The phenotypic effect of this mutation suggests that altering the tesmin domain compromises LIN-54 function and control experiments indicated that LIN-54 protein levels are normal in *lin-54(n2990)* mutant animals (see below). The *lin-54(n2231)* allele encodes a protein that contains an additional change in the C-terminus (A442T) (Figure 1A). We reasoned that these mutant alleles might result in loss of *lin-54* function because the corresponding protein fails to interact with other DRM complex components, because it fails to bind DNA, or because of a combination of these effects.

**Figure 2:**
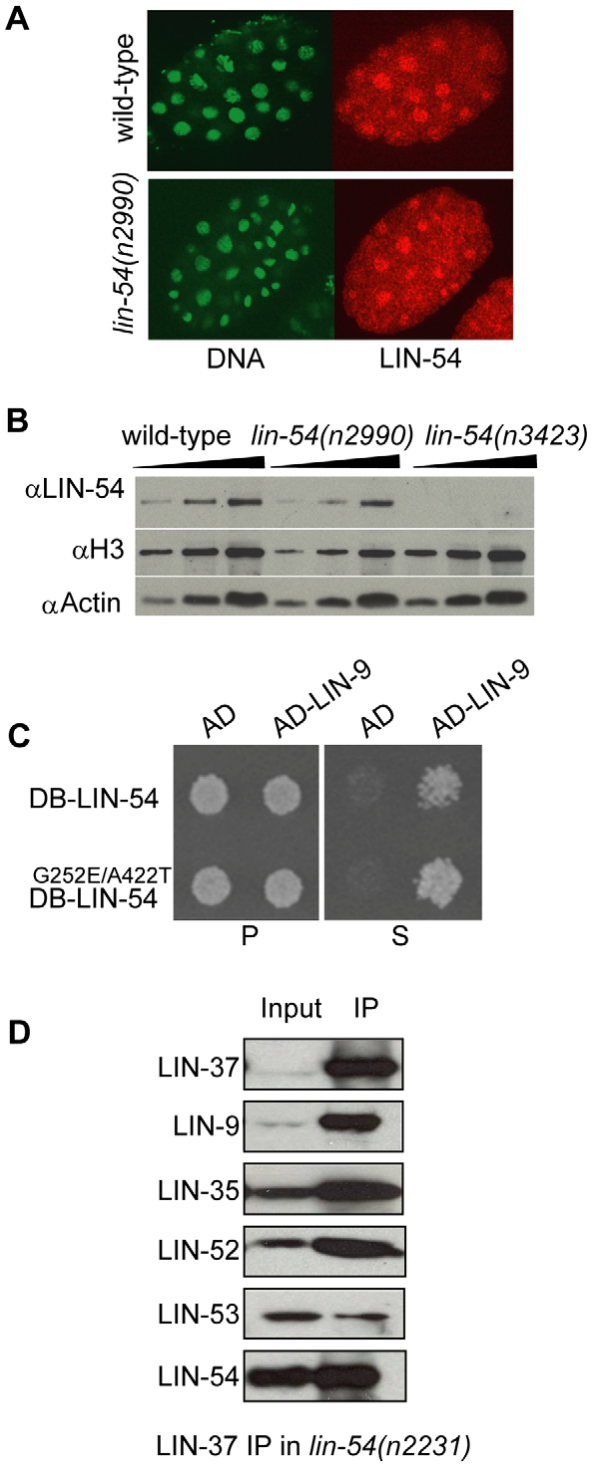
LIN-54 tesmin domain mutation does not disrupt its stability or association with DRM. (*A*) Immunofluorescence of LIN-54 in embryos from wild-type and *lin-54(n2990)* animals. (*B*) Western blots of whole worm extracts from wild-type, *lin-54*(*n2990*), and *lin-54(n3423)* mutants, probed with antibodies against LIN-54, histone H3, and actin. Lanes contain protein from 25, 50, and 100 worms. (*C*) Yeast two-hybrid assay using either wild-type LIN-54 (top) or mutant LIN-54^G252E/A442T^ (bottom) as bait and LIN-9 as prey. DB = Gal4 DNA-binding domain. AD = Gal4 activation domain. P = permissive media, S = selective media. (*D*) Immunoprecipitation using antibodies against LIN-37 in *lin-54(n2231)* tesmin mutant extract, and probed with antibodies listed at left.

Previously, we found that LIN-54 can bind multiple *C. elegans* gene promoters in Y1H assays [22]. To ask whether the tesmin domains mediate DNA binding, we tested wild-type LIN-54, and mutant versions of LIN-54 carrying lesions in a single tesmin domain (G252E and G252E/A442T), or lesions in both tesmin domains (K186E/G252E) in Y1H assays. We found that the mutant proteins exhibited much weaker DNA binding compared to the wild-type protein (Figure 1A and 1B). To examine the function of the tesmin domains in DNA binding *in vivo*, we performed chromatin immunoprecipitation (ChIP) experiments with wild-type and *lin-54(n2990)* mutant animals. Because we had noticed that LIN-54 binds its own promoter (Figure 1B), as well as promoters of genes encoding other DRM subunits (Figure S1B), we assayed binding at the *lin-9* and *lin-54* promoters. We observed a 4- and 2-fold decrease in LIN-54 binding in the *lin-54(n2990)* mutant relative to wild-type animals at promoters of *lin-9* and *lin-54*, respectively (Figure 1C, Figure S1C, p-value<0.01). Furthermore, the binding of other DRM complex proteins was also greatly reduced in *lin-54(n2990)* mutant animals (Figure 1C, p-value<0.01). These findings were supported by immunofluorescence analysis, which showed reduced chromosome localization of several DRM complex proteins in *lin-54(n2990)* mutant germlines (Figure S1D). Control experiments showed that wild-type and *lin-54(n2990)* mutant animals produce a comparable amount of full-length, nuclear-localized LIN-54 protein (Figure 2A and 2B), unlike *lin-54(n3423)* null animals which produce no detectable LIN-54 protein and reduced amounts of other DRM subunits (Figure 2B and [4]). Together, these results indicate that LIN-54, in addition to EFL-1/DPL-1 (E2F/DP), is a DNA binding protein involved in recruiting the DRM complex to its target genes.

### LIN-54 Tesmin Domain Mutations Do Not Disrupt DRM Complex Formation

We next tested whether LIN-54 tesmin mutations affect DRM complex formation in addition to compromising DNA binding. Using yeast two-hybrid assays, we found that both wild-type and mutant LIN-54 proteins can interact with the DRM subunit LIN-9 (Figure 2C). In addition, other DRM complex members co-precipitated in *lin-54(n2231)* mutant animals (Figure 2D). These observations demonstrate that the tesmin mutation does not result in an unstable protein and does not compromise the integrity of the DRM complex. We conclude that the *lin-54* tesmin mutant phenotypes are most likely caused by a defect in DNA binding.

### LIN-54 Binds Genes Involved in Development, Reproduction, and Cell Division

**Figure 3:**
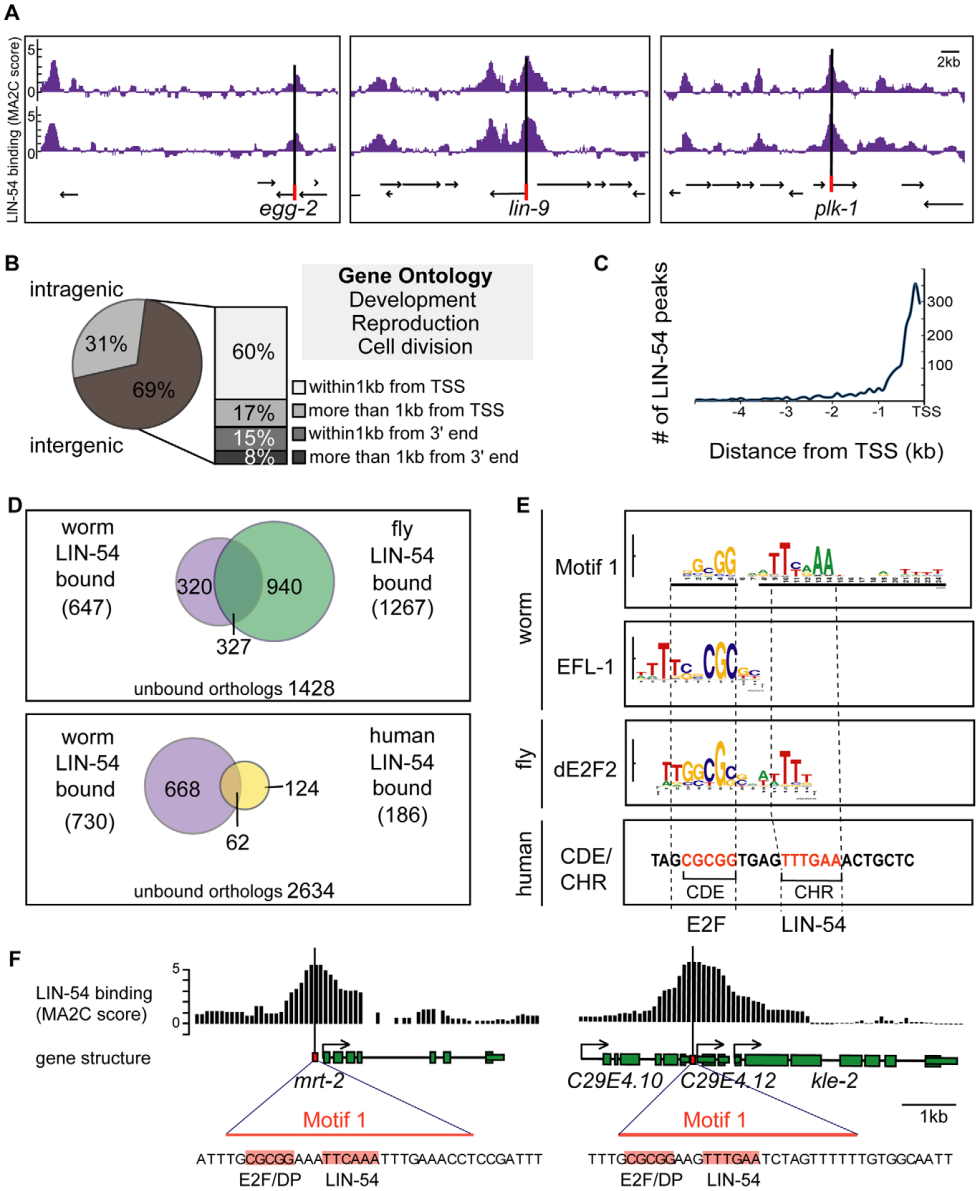
LIN-54 binding is enriched at promoters of genes involved in development, reproduction, and cell division that contain a putative E2F-LIN-54 binding motif. *(A)* Representative MA2C derived peaks from two biological replicates of LIN-54 ChIP-chip from mixed-stage worms. Arrows indicate genes and direction of transcription. *(B)* Relative locations of LIN-54 ChIP peaks. The distance between the mode of each LIN-54 ChIP peak and the translational start site (TSS) of neighboring genes was calculated, and the percentages of four classes of LIN-54 locations are indicated. Enriched gene ontology (GO) terms among genes with peaks within 1 kb of their TSS include development, reproduction, and cell cycle/cell division. *(C)* The numbers of intergenic LIN-54 peaks relative to their distance from the nearest TSS. *(D)* Conservation of orthologous LIN-54 binding targets between worms, flies, and humans. (*E)* An overrepresented motif in LIN-54-bound promoters (Motif 1, top). Aligned below are previously defined motifs: the *C. elegans* EFL-1 consensus [33], an extended *Drosophila* dE2F2 motif enriched among dE2F2, dLIN-9 and dLIN-54 co-regulated genes [21] and the human CDE/CHR motif from the cdc2 promoter [23]. Dotted lines outline regions bound by human E2F4 and LIN-54 at cdc2 and their homologous motif sequences in other organisms. *(F)* Examples of LIN-54 binding (ChIP peaks shown by black bars representing MA2C score) and location of Motif 1 (orange square) at promoters of two genes (*mrt-2* and C29E4.12, arrows = TSS; green boxes = exons).

We used ChIP-on-chip to identify genomic regions bound by LIN-54 in mixed-stage wild-type animals. Reproducible peaks of LIN-54 binding were detected in two biological replicas by the program MA2C (model-based analysis of two-color arrays, Figure 3A) [32]. Using the MA2C criteria described in Materials and Methods, we identified 1992 LIN-54 binding peaks (Table S1). We used the mode of each peak as a measure for the location of LIN-54 association and found that 69% of the regions bound by LIN-54 occur within intergenic regions (Figure 3B). We next determined the relative position of intergenic LIN-54 peaks with respect to surrounding genes. We found that 60% of intergenic LIN-54 peaks occur within 1 kb upstream of protein-coding genes, and that the occurrence of a LIN-54 peak dramatically declined with distance from the translational start site (Figure 3B and 3C). When transcription factors bind between divergently transcribed genes it is difficult to determine whether they regulate one or both genes, so in these cases we considered the binding to be associated with both adjacent genes. Overall, LIN-54 bound to 1572 protein-coding gene promoters (Table S1). These genes are highly enriched for three major gene ontology (GO) branches: developmental process (p-value<10^−100^), reproduction (p-value<10^−100^), and cell division (p-value<10^−30^) (Table S1). These results agree with and extend observations of DREAM function in *Drosophila* and human tissue culture cells [5], [21] and show that DRM has conserved roles in development.

### LIN-54 Target Genes Are Conserved through Evolution

We discovered a significant degree of overlap among the individual genes bound by LIN-54 in worms, flies and humans (Figure 3D). The HomoloGene program has compared *D. melanogaster* and *C. elegans* genomes and defined a total of 3015 orthologous gene pairs (see Materials and Methods). Restricting our analysis to these defined fly-worm ortholog pairs, we note that 1267 are bound by LIN-54/Mip120 in flies [21], 647 are bound by LIN-54 in worms (this study), and 327 are bound in both species (p-value<10^−6^). Commonly bound genes are enriched for developmental GO terms such as sex differentiation as well as cell division terms such as cytokinesis and cell cycle (Table S1). Commonly bound orthologs are involved in multiple aspects of cell division (*smc-3*, *zyg-9*, *air-2*, *plk-1*, *cye-1*), DNA replication and repair (*cdc-6*, *mcm-2*, *pri-1*, *mre-11*, *rad-51)* and transcription and chromatin regulation (*rbp-6*, *taf-4*, *mys-1*, *ash-2*, *mrg-1*). We also found significant overlap of genes bound by worm and human LIN-54: 62 orthologous gene pairs are bound in both species (p-value<10^−4^, Figure 3D) [5]. Further, in all three species, DREAM binds immediately upstream of genes in proximal gene promoters (this study; [5], [21]). Thus, LIN-54 targets the DREAM complex to genes involved in similar overall biological processes in three different phyla by binding to the proximal promoters of multiple orthologous genes.

In all three species DREAM bound the promoters of genes encoding its own subunits. (Figure 1B and 1C, Figure 3A, Table S1, Figure S1B) [5], [21]. *C elegans* LIN-54 also bound the promoters of other synMuvB class genes, including LIN-61/L(3)MBT, LIN-15B, LIN-13, and LET-418 (Table S1). This may suggest conserved transcriptional feedback between DRM subunits and perhaps other synMuvB class genes. However, genes encoding DREAM subunits show little change in expression upon LIN-54 depletion in *D. melanogaster* or *C. elegans* ([21], Table S2, data not shown). Perhaps the effects of DREAM autoregulation are small and required only to buffer DREAM levels and function.

### A Hybrid E2F/DP and LIN-54 Putative Binding Motif

We identified two DNA motifs that are over-represented in LIN-54-bound promoters in *C. elegans* (Figure 3E, Figure S2). Motif 1 appears to be a hybrid E2F/DP and LIN-54 motif (Figure 3E, top) and is usually found near the center of LIN-54 ChIP peaks (Figure 3F and Figure S2). The 5′ end of this motif is similar to previously reported E2F/DP binding sites in *C. elegans* and other organisms ([25], [33], [34], http://jaspar.genereg.net). The 3′ end of Motif 1 resembles a *cis*-regulatory element in the human *cdc2* promoter (called CHR, or cell cycle homology region), which can be directly bound by hLin54 *in vitro* [23]. E2F/DP binding sites co-occur with CHRs in the promoters of some human genes, with a similar orientation and spacing as the motif we identified here ([34], Figure 3E “human”). Moreover, a related motif was identified from *Drosophila* DREAM-regulated genes ([21], Figure 3E “fly”). These results suggest conserved recruitment of the DREAM complex to its target genes by two DNA binding moieties: EFL-1/DPL-1 (E2F/DP) and LIN-54. LIN-54 bound promoters were also enriched for a periodic T-rich motif that resembles a related motif in *Drosophila* DREAM-bound genes (Motif 2, Figure S2, [21]). Other examples of periodic T-rich promoter motifs include sequences that function as nucleosome positioning signals [35] and elements with unknown function that are enriched in *C. elegans* germline-expressed promoters [36].

### LIN-54 Can Activate or Repress Gene Expression

Mutations in *lin-54* confer both germline and somatic abnormalities ([4], [11], Figure S3). To identify genes regulated by LIN-54 *in vivo*, we performed microarray expression profiling analysis of wild-type and *lin-54* mutant *C. elegans* embryos and of isolated germlines. We chose embryos because they consist primarily of somatic cells, at a developmental stage with both active cell divisions and dynamic developmental gene expression programs. Since *lin-54* null animals are sterile [4], embryos were obtained from the *lin-54(n2990)* strain. *lin-54(n2990)* is a partial loss-of-function allele that causes the same spectrum of phenotypes as a null allele, albeit weaker, making it an appropriate strain in which to examine partial loss of *lin-54* function ([4], Figure S3A). Germlines were dissected from *lin-54* null adults that lack detectable *lin-54* transcript and protein ([4], Figure 2, and data not shown), exhibit reduced levels of other DRM complex proteins [4], and exhibit reduced germline chromosome association of DRM complex proteins tested (Figure S1D). We isolated the germline region from the tip until late pachytene stage of meiosis, because nuclei in this region are morphologically similar between wild-type and mutant (Figure S3B) and are undergoing X chromosome silencing [26]. While embryos contain a few primordial germ cells and dissected germlines contain some cells of the somatic gonad, the two samples predominantly represent somatic and germline tissue, respectively.

**Figure 4:**
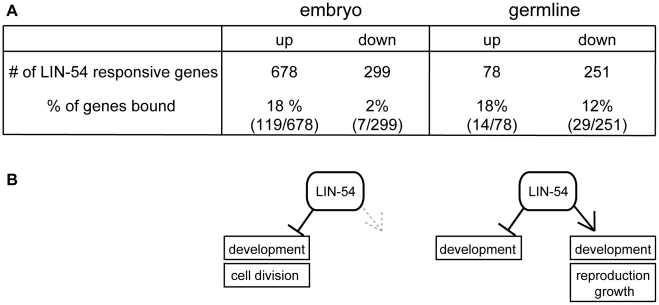
LIN-54 can function as a transcriptional activator or repressor. (*A*) Microarray gene expression profiling analysis of *lin-54(n2990)* embryos and *lin-54(n3423)* germlines. Genes that change expression in *lin-54* mutant animals are grouped into four classes: “up in embryo”, “down in embryo”, “up in germline” and “down in germline”. Overlap with LIN-54 ChIP peaks is indicated. (*B*) Cartoon indicating the inferred regulation by wild-type LIN-54 in embryo (left) or germline (right) and the major Gene Ontology (GO) terms associated with each class of regulated genes. p-value<0.05 for all GO terms.

We identified 678 genes whose transcripts increased at least 1.5-fold in mutant embryos (Figure 4A, Table S2). Of these, 119 (18%) were also bound by LIN-54 (Figure 4A). We note that ChIP was performed on mixed-stage animals to survey binding sites, while microarray was performed on a single stage, which may make it more difficult to identify all genes that are both bound and regulated. Nevertheless, this degree of overlap is similar to that observed in other ChIP and microarray studies [21], [37], and suggests that this gene set includes direct targets bound and regulated by LIN-54. GO analysis of up-regulated genes or of bound and up-regulated genes revealed over-represented terms related to development (Table S2), terms that were also enriched among genes bound by LIN-54 (Table S1). Fewer genes showed reduced expression in mutant embryos (299, Figure 4A). These genes showed no GO term overlap with LIN-54 bound genes, and only 2% (7/299) contained LIN-54 ChIP peaks at their promoters. This observation suggests that most of these genes are regulated indirectly. We conclude that LIN-54 predominantly functions as a transcriptional repressor in embryos (Figure 4B).

We noted that many up-regulated genes fell into discrete functional sub-categories related to development. Some of these gene sets might explain abnormalities of synMuvB mutant animals. For instance in *lin-54* mutant embryos, 18 up-regulated genes are involved in meiosis (GO term GO0001726) and overall, 11% of the up-regulated genes normally show germline-specific or enriched expression [38]. Previously, mutations in synMuvB genes were shown to cause ectopic expression of certain germline P granule components in the soma, proposed to reflect soma to germline transformation [18], [39]. Our genome-wide study strengthens this model by indicating that LIN-54 represses transcription of a variety of germline genes in embryo soma, including the P granule protein *glh-1*, the meiotic recombination protein *spo-11*, and the eggshell protein *cpg-2*. We also observed up-regulation of many RNA interference pathway genes in *lin-54* mutant embryos, including *ego-1*, *rde-4*, and *sago-2*. If these factors are normally limiting for a full RNAi response, their up-regulation might account for the enhanced RNAi phenotype that has been observed in synMuvB mutants [17], [18].

In the germline, 78 genes showed increased and 251 genes showed decreased expression in mutant relative to wild-type animals (Figure 4A, Table S2). Both sets of genes exhibit overlap with LIN-54 ChIP peaks (18% and 12%, respectively) (Figure 4A). Further, both up-regulated and down-regulated germline genes are enriched for development GO terms, which again overlaps with the terms found in the ChIP data (Figure 4B, Table S2). These observations suggest that both up- and down-regulated germline genes could include targets directly regulated by LIN-54. While the development GO term is associated with both embryonic and germline LIN-54 target genes, reproduction and growth terms were only enriched in genes with decreased expression in the *lin-54* mutant germline. These reproduction genes that we presume are normally activated by LIN-54 include germline-produced transcripts required for meiosis, oogenesis and early embryogenesis, as observed previously for EFL-1/DPL-1 [25]. Thus in contrast to embryos, in the germline LIN-54 appears to both activate and repress gene expression, and activates a distinct set of reproduction and growth genes required for germline function.

### LIN-54 Binding Is Under-Represented on the X Chromosome

**Figure 5:**
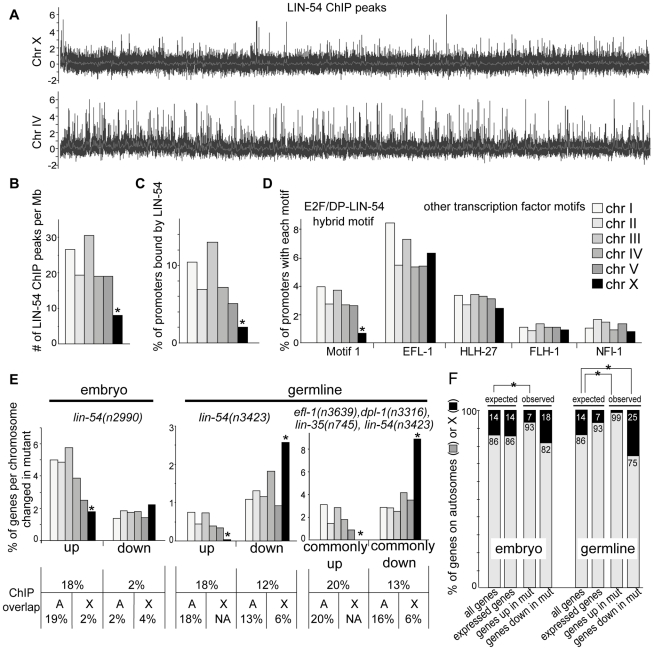
LIN-54 shows autosome-enriched binding and chromosome-biased gene regulation. *(A)* LIN-54 ChIP peaks along the entire X chromosome (top) and chromosome IV (bottom). *(B–C)* Number of LIN-54 ChIP peaks per mega base *(B)* and percentage of promoters bound by LIN-54 *(C)* on each *C. elegans* chromosome. LIN-54 ChIP peaks occur less frequently on the X chromosome, independent of chromosome size and gene density. *(D)* Occurrence of putative E2F/DP-LIN-54 binding Motif 1 and other transcription factor binding motifs in promoter regions (1 kb upstream from translational start site) of autosomal genes and X-linked genes. Motif 1 is under-represented in X-linked promoters. *(E)* Chromosome distribution of genes up-regulated or down-regulated in *lin-54(n2990)* embryos (left), *lin-54(n3423)* germline (middle), or commonly co-regulated by cluster analysis of *lin-54(n3423)*, *efl-1(n3639)*, *dpl-1(n3316)*, *and lin-35(n745)* germlines (right). Overlap with LIN-54 ChIP peaks for an average autosome or X chromosome is indicated below. Commonly up is group E, commonly down is group B from Figure S4. Data for *efl-1*, *dpl-1*, and *lin-35* are from [25]. *(F)* The percentage of genes located on the five autosomes (gray) or the X chromosome (black). Expected values are presented both for all genes in the genome, and for all genes normally expressed (expressed genes) in embryo or germline, and compared to observed percentages of genes up-regulated (genes up in mut) or down-regulated (genes down in mut) in *lin-54* mutants. Asterisks indicate p-value<10^−3^ by Fisher's Exact test orG-test.

We discovered a striking non-uniform distribution of LIN-54 binding across the *C. elegans* genome: X chromosomes had significantly fewer LIN-54 ChIP peaks than autosomes (p-value<10^−15^, Figure 5A). Each autosome had on average 369 LIN-54 ChIP peaks (23 peaks per Mb), whereas the X chromosome contained only 145 (8 peaks per Mb) (Figure 5B, Table S3A). On average, 8% of autosomal gene promoters, but only 2% of X chromosome promoters, were bound by LIN-54 (Figure 5C, Table S3A, p-value<10^−41^). This analysis shows that LIN-54-bound promoters are significantly under-represented on the X chromosome, independent of chromosome size and gene density.

We also found that the hybrid motif (Motif 1, Figure 3E), as well as the T-rich motif (Motif 2, Figure S2A), were under-represented on X compared to autosome promoters (Figure 5D, Figure S2B, Table S3B, p-value<10^−13^ for Motif 1). However, a published EFL-1 consensus site alone shows no bias against X chromosomes (Figure 5D, [33]). A uniform distribution was also observed for three additional transcription factors for which a consensus DNA binding motif has previously been determined (HLH-27, FLH-1, and NFI-1, Figure 5D) [40]–[42]. These results imply that the DRM complex is recruited more frequently to autosomes than to the X chromosome through the combined DNA binding activities of LIN-54 and EFL-1.

### *lin-54* Mutants Exhibit Chromosome-Biased Gene Expression Changes

We addressed whether the non-uniform binding of LIN-54 in the genome results in differential regulation of autosomal versus X-linked genes. LIN-54-responsive genes are distributed across all six *C. elegans* chromosomes (Table S3), and we analyzed chromosome bias in two ways. First, to normalize for the variable number of genes on each chromosome, the percentage of LIN-54 responsive genes out of all genes per chromosome was calculated (Figure 5E). Second, to compare expected to observed distributions, we calculated the percent of all genes in the genome located on autosomes and compared that to the percent of LIN-54 responsive genes on autosomes (Figure 5F “all genes” versus “genes up in mut” or “genes down in mut”). Additionally, because the germline has an inherent autosomal bias in its expressed genes, we also calculated the percent of autosomal genes typically expressed in embryo or in germline as “expected” and compare that to the “observed” percent of LIN-54 responsive genes that reside on autosomes in each sample (Figure 5F “expressed genes” versus “genes up in mut” or “genes down in mut.”

Embryonic genes that were up-regulated in *lin-54* mutants are over-represented on autosomes (633/678, 93% observed versus 86% expected by chance, p-value<10^−8^, Figure 5E and Figure 5F “embryo up”). This finding is consistent with the idea that LIN-54 is preferentially recruited to autosomes, and primarily acts as a repressor in the embryo. Embryonic genes down-regulated in *lin-54* mutants showed no significant chromosomal bias, consistent with our interpretation that these genes are mostly indirectly regulated (244/299, 82% versus 86% expected by chance, p-value = 0.03, Figure 5E and Figure 5F embryo down).

To our surprise, LIN-54 exhibited two different patterns of chromosome-biased gene regulation in the germline. Genes up-regulated in *lin-54* mutants were over-represented on autosomes, to a degree that is significantly different from all genes (77/78, 99% versus 86% expected by chance for all genes, p-value<10^−3^, Figure 5E and Figure 5F), and comparable to the inherent bias of the germline (99% versus 93% expected by chance for germline-expressed genes, p-value = 0.06). This is consistent with the autosome-biased localization of LIN-54. LIN-54 is likely a direct repressor of at least some of these genes, since 18% overlap with LIN-54 ChIP peaks (Figure 5F). In striking contrast, germline genes that were down-regulated in *lin-54* mutants were located more frequently on the X chromosome than expected (64/251, 25% versus 14% expected by chance for all genes, p-value<10^−5^, or versus 7% expected by chance for all germline-expressed X-linked genes, p-value<10^−40^, Figure 5E and Figure 5F, “germline down”).

It appears paradoxical that LIN-54 and its binding motif are preferentially located within autosomal gene promoters, yet in the absence of LIN-54 more genes on the X chromosome than on an average autosome decrease expression in the germline. One possibility is that LIN-54 affects these X-linked genes indirectly, which would predict less correlation between binding (ChIP peaks) and gene expression changes. Indeed, down-regulated X-linked genes overlap less frequently with LIN-54 ChIP peaks than down-regulated autosomal genes (6% versus 13% overlap, Figure 5E). Our interpretation of this observation is that LIN-54 is normally a direct activator of at least some autosomal genes that are down-regulated in the mutant, but that LIN-54 more indirectly regulates X-linked genes. Perhaps LIN-54 regulates an autosomal gene involved in X chromosome gene regulation, or prevents inappropriate spread of a repressor to the X chromosome (see Discussion).

Another apparent paradox is that LIN-54 loss leads to down-regulation of X-linked genes, when X chromosomes already undergo chromosome-wide silencing in the hermaphrodite germline. However, when we examined transcripts normally expressed in our wild-type germline samples using “present” calls from microarrays, we found that 15% of all X-linked genes are in fact expressed (376/2491 on array), consistent with published estimates from SAGE analysis (Materials and Methods, [38]). Of the 376 total germline-expressed X-linked genes, 17% are down-regulated in the *lin-54* mutant (64/376) while only 4% of all germline-expressed autosomal genes are down-regulated (187/5097). The large percentage of total X-linked genes affected in the mutant may support models in which LIN-54 has chromosome-wide effects on X chromosome transcription (see Discussion). Thus on the X chromosome, the loss of LIN-54 function causes further silencing of X-linked genes.

### The DRM Complex Preferentially Localizes to Germline Autosomes

We wondered whether the chromosome-biased localization and function of LIN-54 are features shared by other members of the DRM complex. We first compared germline expression profiles of *lin-54(n3423)* with published germline expression profiles for *efl-1(n3639)*, *dpl-1(n3316)*, and *lin-35(n745)* mutant animals ([25], Figure S4). Genes commonly down-regulated in all four DRM mutants were more frequently located on X chromosomes than autosomes, consistent with observations in the *lin-54* mutant (Figure 5E, “commonly down” and Figure S4, group B). Also consistent was the finding that commonly down-regulated X-linked genes overlapped less frequently with LIN-54 ChIP peaks than commonly down-regulated autosomal genes, again suggesting that more X-linked genes are regulated indirectly (6% versus 16% overlap, Figure 5E). Up-regulated genes common to all four mutants were more difficult to define. However, we did note that a commonly up-regulated group of genes primarily regulated in *lin-54(n3423)* (Figure 5E “commonly up”, Figure S4 group E) and another cluster primarily up-regulated in *lin-35(n745)* (Figure S4 group I) were each autosome-enriched, as observed for the *lin-54* mutant alone. These results show that similar patterns of chromosome-biased gene regulation are exhibited by multiple DRM subunits.

**Figure 6:**
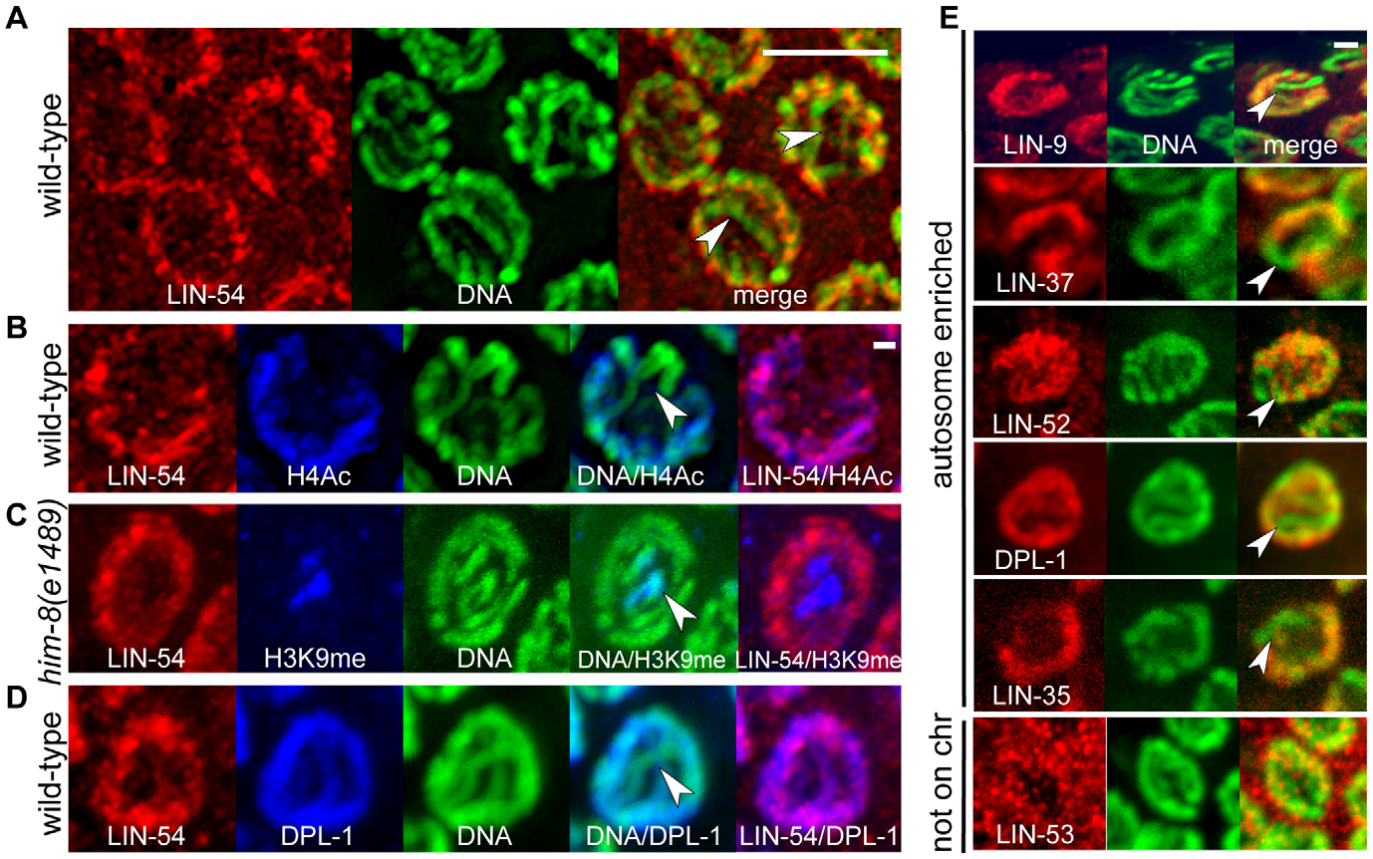
DRM complex members localize to germline autosomes. Shown are nuclei in the meiotic pachytene stage in the hermaphrodite germline. Arrowheads indicate a chromosome in the nucleus with less LIN-54 staining *(A–D)* or less staining of other DRM subunits *(E)*. *(A)* Immunofluorescence with anti-LIN-54 antibody (red) and DNA dye (green, merge in yellow). (*B*) Antibodies against a histone modification associated with active transcription (H4K12Ac, blue) show enrichment on autosomes, and co-localize with LIN-54 (red, DNA in green). (*C*) LIN-54 (red, DNA in green) staining in the *him-8(e1489)* mutant in which X chromosomes do not pair and acquire the histone modification H3K9me2 (blue). *(D)* Co-staining of LIN-54 (red) with DPL-1 (blue, DNA in green). Both are under-represented on the X chromosome (arrowhead). *(E)* Immunofluorescence of DRM complex subunits (red) on wild-type germline nuclei (DNA, green; merge yellow). Images in *A* and *B* represent deconvolved confocal stacks. Scale bar represents 5 µm (A) or 1 µm (B–E).

Next, we examined the chromosomal localization of DRM complex members in the germline by immunofluorescence. Figure 6 shows nuclei in the pachytene stage of meiotic prophase, when homologous chromosomes are paired and beginning to condense. LIN-54 (red) co-localized with DNA (green), with the exception of one prominent region (Figure 6A, arrowheads). We demonstrated that this region corresponds to the X chromosome in two different ways. First, LIN-54 colocalized with H4K12Ac (blue), a histone modification associated with actively transcribed regions, which is under-represented on the partially silenced X chromosome ([26], Figure 6B). Second, LIN-54 did not co-localize with the H3K9me2-stained X chromosome in *him-8(e1489)* mutants (Figure 6C). In these mutants the X chromosomes do not pair during meiosis and therefore acquire this heterochromatic histone mark [43].

The DRM complex members LIN-9, LIN-35, LIN-37, LIN-52, and DPL-1 were also under-represented on the X chromosome in the germline (Figure 6D and 6E). Thus, most DRM complex members localize on autosomes. Only one DRM subunit was not autosome-enriched. The CAF1 homolog LIN-53, which participates in multiple complexes [4], showed little localization to DNA during this stage of meiotic prophase (Figure 6E). It is interesting to note that despite the uniform genomic distribution of the EFL-1/DPL-1 motif, DPL-1 was enriched on the autosomes in the germline and co-localized with LIN-54 (Figure 6D). These results support the hypothesis derived from our motif analysis that when EFL-1/DPL-1 and LIN-54 jointly bind Motif 1, this complex disfavors the X chromosome. These results are also consistent with the finding that germline genes co-regulated by EFL-1/DPL-1 and LIN-54 share similar biases in chromosome location. We conclude that LIN-54 acts with other DRM complex members to govern chromosome-biased gene regulation in *C. elegans*.

## Discussion

Our genome-scale analyses of LIN-54 provide new insights into the binding and regulatory activities of the conserved transcription factor complex DRM. Our results in *C. elegans*, considered along with those available from *Drosophila* and human cells, highlight both conserved and non-conserved features of DRM. Conserved features include 1) DRM recruitment to promoters with a hybrid E2F/DP and LIN-54 binding motif, likely by the coordinated action of LIN-54 and E2F/DP, 2) its regulation of genes involved in cell cycle, development, and reproduction, and 3) its activity as both an activator or repressor. Through analysis of cells from a developing organism, we revealed conserved critical roles for DRM during animal development and showed that DRM activities vary in different tissues. Remarkably, we found that DRM binding and regulation are chromosome-biased in *C. elegans* but not *Drosophila* or humans, perhaps due to evolutionary pressures imposed by X chromosome silencing mechanisms.

### Targeting the DREAM Complex to Promoters

Several members of the DREAM transcription factor complex have known or presumed DNA binding activity, but how they act in concert to direct promoter recognition was not well understood. Here we show that the DRM component LIN-54 binds DNA directly, helps recruit DRM to promoters *in vivo*, and likely recognizes a hybrid E2F/DP and LIN-54 consensus motif. In *Drosophila* and humans, Myb is a DNA-binding component of the DREAM complex and it has been shown that Myb and E2F/DP function in a mutually exclusive manner [5]–[7], [21]. We show that LIN-54 is another key DRM recruitment subunit and may function coordinately with E2F/DP: the E2F/DP and LIN-54 motifs co-occur in LIN-54 target genes and both components regulate a common set of genes. Our recognition that the *C. elegans* hybrid Motif 1, the CDE/CHR element of human cell cycle genes, and a motif identified in *Drosophila* DRM-bound genes are related elements suggests that coordinate binding by E2F/DP and LIN-54 is a conserved means of recruiting DRM to promoters (this study, [5], [21], [34]). It has been observed that the E2F binding motif is more widely distributed than E2F family protein binding in *vivo*, and E2F family members often rely on cooperating transcription factors bound to neighboring sites for specificity [44]. Simultaneous binding of adjacent sequence motifs by E2F/DP and LIN-54 might increase the affinity of DREAM for target sites and might provide increased selectivity for target gene recognition. Future studies will reveal if there is a Myb-like component in the *C. elegans* DRM complex, and whether other subunits contribute to DRM targeting to the genome.

### Conservation of DREAM Function

Genes bound and regulated by *C. elegans* LIN-54 predominantly function in development and differentiation, cell cycle and cell division, and in reproduction. Similar categories of regulated genes have been reported in genome-wide studies of *Drosophila* DREAM [21]. In human tissue culture cells, however, only cell cycle genes were enriched [5], [7]. The similarities between *C. elegans* and *Drosophila* suggest broad conservation of DREAM function in both cell cycle and developmental gene regulation. Within the common GO term categories targeted by the DREAM complex, interesting functional subcategories were conserved. In all three organisms DREAM binds groups of genes involved in cell division processes such as sister chromatid cohesion, spindle assembly, and cytokinesis, as well as DNA replication and DNA repair. Both worm and fly DREAM bind and regulate genes involved in sex differentiation such as those required for genitalia formation, and genes required for germline functions including gametogenesis, fertilization, and meiosis. It seems likely that DREAM also regulates transcription of developmental and reproduction genes in mammalian systems, given known developmental roles of its individual subunits and the overall conservation of DREAM function. Perhaps developmental genes were not observed in mammalian studies because of the use of cultured cells derived from differentiated tissues. We find that similarities of DREAM function across species lie not only at the overall level of biological processes: a remarkable degree of overlap exists among individual target genes. Further, the genes targeted by DREAM in all these organisms possess highly similar over-represented E2F/DP-LIN-54 motifs. Altogether, our results unveil an evolutionarily conserved mode of DNA binding that targets the DREAM complex to similar sets of functionally coherent target genes.

### Different Activities of DRM in the Soma and Germline

We demonstrate that DRM acts differently in the soma versus the germline. In embryos, LIN-54 appears to primarily repress genes (a majority of genes are up-regulated in the mutant, and up-regulated genes overlap with LIN-54 ChIP peaks and ChIP GO terms). In the germline, LIN-54 appears to primarily activate genes, yet may also serve as a repressor (a majority of genes are down-regulated in the mutant, and both up- and down-regulated genes overlap with ChIP peaks and ChIP GO terms). The target genes regulated in embryo versus germline are largely distinct, and fall into different enriched functional pathways (Figure 4, Table S2). For example, in the germline LIN-54 promotes expression of genes required for germline functions like oogenesis, meiosis, and fertilization, as observed previously for EFL-1 and DPL-1 [25]. In the embryo, however, LIN-54 does just the opposite: it represses germline-specific genes to prevent their ectopic activation in the soma. Even patterns of chromosome-biased gene regulation mediated by LIN-54 showed differences between soma and germline, as discussed below. Our results highlight how DRM may serve as either an activator or repressor. The mechanisms by which DRM may either activate or repress gene expression are at present not well understood, but may involve sub-complexes with different subunit composition or interactions with transcriptional co-factors such as chromatin modifiers. Importantly, our results provide the first genome-wide comparison of DRM function in two cell types isolated from whole animals, and indicate that DRM function differs depending on developmental context. Continued genome-wide analyses of DREAM binding and regulation in a variety of organisms, particularly using specific tissues isolated from animals, will further our understanding of how this key transcriptional complex functions during development and reproduction.

### Why Does *C. elegans* DRM Avoid X Chromosomes?

We discovered that *C. elegans* LIN-54 binding and gene regulation are autosome-enriched. This bias is likely a feature of the worm DRM complex as a whole, since the localization patterns of all but one DRM subunit are autosome-enriched, as are a class of germline genes co-regulated by multiple DRM subunits. Biased binding appears to be directed by a biased recruitment element, since the hybrid E2F/DP-LIN-54 recognition motif is also autosome-enriched in *C. elegans*. However, when we examined the related hybrid motif in *Drosophila* (Figure 3E “fly”), and the published *Drosophila* and human DREAM ChIP profiles we found that they are evenly distributed between autosome and X chromosome promoters (data not shown, [5], [21]).

What evolutionary pressures might have driven the *C. elegans* DRM complex to disfavor the X chromosome? X chromosomes differ from autosomes in many aspects including histone variants and modifications, gene regulation, and rates of gene divergence and movement [45]. One possibility is that DRM targets are under-represented on the X chromosome because some aspect of this chromosomal environment is incompatible with DRM-mediated transcription regulation. A second possibility is that DRM localization and its differential regulation of autosomal and X-linked genes reflects some role in balancing autosome and X chromosome gene expression. Only a limited number of non-histone proteins have been shown to exhibit X chromosome- or autosome-biased localization, and these are involved in somatic dosage compensation or germline X chromosome silencing [46]–[48]. A third possibility is that the biased localization of DRM arose as a consequence of X chromosome silencing in the germline. The X chromosome is silenced in the germline by mechanisms that are distinct from somatic X chromosome silencing [27]. Germline-expressed genes and genes with essential functions are autosome enriched, and thought to have “fled” the X chromosome to avoid being silenced [28]–[30], [49]. One hypothesis is that the DNA-binding properties of the *C. elegans* DRM complex co-evolved with the redistribution of its germline-expressed and essential target genes across the genome, resulting in an autosomal bias. Silencing of the X chromosome has not been reported in *Drosophila* or mammalian female germlines, perhaps explaining why autosome bias is specific to *C. elegans* DRM. The regulation of sex chromosome gene expression, by processes that evolve rapidly and vary widely among organisms, may therefore have consequences on the genomic distribution of gene sets and, as shown here, their transcriptional regulatory networks.

### A Paradox in Chromosome-Biased Gene Regulation

In embryos, the biases in DRM localization and DRM-mediated regulation correspond, but in the germline they do not. In *lin-54* mutant embryos, up-regulated genes likely include direct targets based on their overlap with LIN-54 ChIP peaks, and were autosome-enriched like DRM binding. The down-regulated genes, on the other hand, are more likely indirect targets and showed no chromosome bias. In *lin-54* mutant germlines, both up- and down-regulated genes included direct DRM targets. As in embryos, the up-regulated genes in the germline were primarily autosomal. Interestingly, down-regulated germline genes were X-enriched.

How can we explain the paradox that the DRM complex predominantly binds to autosomes, but that its loss results in a decrease in expression of X-linked genes? First, some LIN-54 does bind the X chromosome and might directly activate gene expression. However, fewer LIN-54-responsive genes on the X chromosome than on an average autosome are bound by LIN-54, suggesting that many X-linked genes are indirectly regulated. Second, loss of LIN-54 might induce ectopic soma-specific pathways that include X-linked genes. However, we found no evidence for enrichment of particular pathways among the affected X-linked genes and none are soma-specific. Other models invoke chromosome-wide alterations in X chromosome gene expression. A third model is that DRM regulates expression along the X chromosome indirectly either by activating a gene involved in X chromosome activation or by repressing a gene involved in X chromosome silencing, so that in mutants the X becomes more silenced. We did not find any obvious candidate for such a factor among mis-regulated genes. Finally, a fourth model proposes that a repressor that is normally concentrated on autosomes, perhaps anchored there by DRM, spreads inappropriately to X chromosomes when DRM function is compromised. If that repressor is limiting, autosomal genes will increase in expression while X-linked genes become repressed, which is in agreement with our observations (Figure 5E). Indeed, such reciprocal gene expression changes have been observed when a limiting domain-specific repressor such as the *S. cerevisiae* SIR proteins spread inappropriately, thereby increasing repression at ectopic locations and diluting repression at their normal site of action [50]–[52]. Related models have been invoked to explain why loss of the autosome bound MES-4 product de-silences germline X-linked genes and to explain why loss of the X chromosome bound Dosage Compensation Complex de-silences somatic X-linked genes and represses some autosomal genes in *C. elegans* [53], [54].

### Opposing Actions of DRM and the Histone Methyltransferase MES-4

MES-4 is an autosome-enriched histone methyltransferase that confers the “active mark” H3K36me [53]. In many biological contexts, *mes-4* and synMuvB genes have opposing functions. For example, mutations in *mes-4* can suppress the defects in vulva development, the increased RNAi and transgene silencing, and the ectopic expression of germline genes in the soma caused by mutations in synMuvB genes [18], [39], [55], [56]. Here we define another process in which *mes-4* and synMuvB mutations have opposite effects. We show that in the hermaphrodite germline LIN-54 is autosome-enriched as is MES-4, but *lin-54* mutants down-regulate while *mes-4* mutants up-regulate X-linked genes.

Bender et al. (2006) proposed that MES-4 indirectly regulates X-linked genes, by repelling a “global repressor” from autosomes and keeping it concentrated on the X chromosome. A possibility is that LIN-54 and MES-4 affect the X chromosome versus autosome distribution of the same repressor, in an opposite manner. A candidate for such a repressor is the *C. elegans* Polycomb Repressive Complex 2 (PRC2), which is composed of MES-2, MES-3 and MES-6. MES-2 is an E(z) homolog that concentrates the H3K27me3 “repressive mark” on the X chromosome in the germline [53], [57]. MES-2/-3/-6 also keeps MES-4 and other active marks restricted to autosomes. Interestingly, it was recently shown that a class of genes repressed by the *Drosophila* DREAM complex is enriched for H3K27me2 and requires E(z) for repression [58]. However, the cytological distribution of H3K27me3 appears unaffected in *mes-4* and *lin-54* mutants ([53], data not shown). An important future direction is to explore potential links between DRM, MES-4, and Polycomb Group mediated gene repression, and to shed light on how these factors might interact to govern gene regulation.

## Materials and Methods

### *C. elegans* Strains and Culture Conditions

All strains were cultured at 20°C unless otherwise noted, using standard methods. The following strains were used: N2 (Bristol) as wild-type, *lin-54(n3423)/nT1 [qIS51]*, *lin-54(n2990)*, *lin-54(n2231)* [4], [11], and *him-8(e1489)* [59]. Note: Previously, *lin-54(n2231)* was reported to have a single mutation (A442T) [4]; however, sequencing revealed an additional missense mutation (G252E).

### Immunofluorescence

Embryos (Figure 2) were fixed with methanol/acetone [60]. Germlines (Figure 6 and Figure S1D) were fixed essentially as described [61], with the addition of 5 ul of 2% Triton-X before fixation in 4% paraformaldehyde. DNA was visualized either with DAPI or OllieGreen (added at 1∶1000 with 10 ug/ml RNAseA with the secondary antibody). Whole worms (Figure S3) were prepared in Carnoy's fixative as described by [62]. Primary antibodies to DRM subunits were described and validated in [4], [10], [13]. Another second anti-LIN-54 antibody was generated in rabbits against amino acids 207–306 (Strategic Diagnostics Inc.), validated by western blot in wild-type and mutants, and showed the same localization patterns. Primary antibodies were used at 1∶100 dilutions, and detected with secondary antibodies conjugated to Alexa Fluor 568 (Invitrogen) at a 1∶500 dilution, except DPL-1 was performed as described [4], [10]. Antibodies against H4K12Ac (Serotec), and H3K9me2 (Cell Signaling) were used at 1∶1000 (primary) and seconday antibodies at 1∶1000. Images for Figure 6 were captured by a Solamere Technology Group modified Yokogawa CSU10 Spinning Disk Confocal scan head attached to a Nikon TE-2000E2 inverted microscope and a 100× Plan Apo objective, using MetaMorph software (Molecular Devices). The images for Figure 6A and 6B were deconvolved using the constrained iterative deconvolution algorithm developed by the UMass Medical School Biomedical Imaging Group [63].

### Yeast One-Hybrid and Two-Hybrid Assays

Y1H and Y2H assays were performed as described [22], [64]. Representative images for Figure 1B were obtained for *Ppos-1* at 10 mM 3AT 5 days, *Plin-54* at 20 mM 3AT 9 days, and *Pvha-15* at 60 mM 3AT 9days.

### Western Blot, Immunoprecipitation, and Chromatin Immunoprecipitation

For western blot (Figure 2B), whole worm lysates were created from 200 hand-picked synchronized young adults boiled in 2× loading buffer (National Diagnostics EC-886) for 30′ with intermittent vortexing. Lysates equivalent to 25, 50, and 100 animals were loaded per lane and probed with anti-LIN-54, actin (Abcam #ab3280, 1∶400) and Histone H3 (Abcam #ab1791, 1∶1000). Immunoprecipitation, western blotting, and probing with DRM antibodies were performed as described [4], ChIP was performed as described [65]. Briefly, mixed stage wild-type worms were cultured in S-basal at 20°C. Lysates were cross-linked in 1% formaldehyde, sonicated, and immunoprecipitated with anti-LIN-54 antibody or pre-bleed antibody control. ChIP samples including the input were subjected to two rounds of linear amplification, using the genomePlex complete whole genome amplification kit (Sigma), and minimum difference between original precipitates and amplified precipitate confirmed by qPCR (data not shown). Both experimental and input were processed at NimbleGen, hybridized on 385K *C. elegans* Whole Genome 3-Array Set (Roche NimbleGen). To assay DRM subunit binding at the promoters of the *lin-9* and *lin-54* genes, ChIP was performed with antibodies against LIN-54, LIN-9, LIN-37, or pre-bleed control from wild-type or *lin-54(n2990)* mixed-stage extracts. qPCR was used to calculate the amount of *lin-54* or *lin-9* promoter DNA in ChIP samples relative to the total input DNA. The ratio in wild-type was set at 1.0. *lin-9* promoter primers: 5′-cgactgtcaaacagcagctc-3′ and 5′-ttgaaatggcggttcttttc-3′. *lin-54* promoter primers: 5′-atgatgagtgacgtctacc-3′ and 5′-attgtttcgcgcgccgaaatttg-3′.

### RNA Isolation and Microarray

#### Embryo

Animals were propagated on egg plates seeded with *E. coli* HB101 at 20°C, bleached to obtain synchronized L1 larvae and then grown at 25°C for 48 hours. Embryos from young adults were harvested by the bleach-alkaline method, and filtered through 100 micron mesh (Small Parts, Inc.). 200 µL of embryo pellet was suspended in 1 mL of Tri reagent (Molecular Research Center, Inc. TR118), flash-frozen, and dounced. Total RNA was purified with RNAeasy mini kits (Qiagen), treated with DNase, and integrity examined on agarose gel.

#### Germline

Animals were grown at 20°C and dissected in 1× egg buffer to excise the germline 24 hours after L4 stage. Germlines were dissected to include mitotic tip through meiotic late pachytene (Figure S3). RNA was isolated as described [25], and linearly amplified once using MessageAmp II aRNA Amplification Kit (Ambion).

#### Microarray

Probe-preparation, hybridization, and scanning for DNA microarray were performed at the Genomics Core facility at University of Massachusetts Medical School. Fluorescence-labeled cDNA probes were prepared using the One-Cycle kit (Affymetrix) and the Enzo HighYield RNA Transcript Labeling Kit (Enzo) for embryo, and the 3′ IVT Express Kit (Affymetrix) for germline. cDNA probes of three replicates were hybridized to GeneChip *C. elegans* genome arrays (Affymetrix).

### Chip Peak Analysis

Raw ChIP-chip data were analyzed using three independent programs: MA2C [32], ChIPOTle [66] and NimbleScan (Roche NimbleGen). While ChIPOTle called fewer and NimbleScan called greater numbers of peaks than MA2C, each identified a similar set of core peaks. MA2C analysis was performed with the following settings: # MA2C Score Method (median), Band Width (300), p-value cut off (−6), and other parameters were set as default. WS180 was used to annotate gene names. LIN-54 ChIP peaks (Figure 3 and Figure 5A) were visualized using Affymetrix Integrated Genome browser. Modes of LIN-54 peaks were used to determine peak location for Figure 3, and each intergenic peak was considered to associate with both neighboring genes.

### Ortholog Pair Analysis

HomoloGene (Ce.01-08-2009) defines 3015 orthologous pairs between *C. elegans* and *D. melanogaster*, and 3488 pairs between *C. elegans* and human. 647 of 1572 genes bound by *C. elegans* LIN-54 have annotated fly orthologs; 730 genes have annotated human orthologs. 1267 of 3147 fly genes bound by Mip120 have worm orthologs (data from Table S3 in [21], using genes bound by Mip120 within 1 kb of 5′ end, lr peak = 2.). Of 975 human genes bound by hLIN54 (data from Table S4 in [5], using genes bound by hLIN54 within 1 kb of 5′ end, during G0 and/or S phase), 186 have annotated worm orthologs.

### Motif Analysis

To predict motifs enriched in LIN-54 bound promoters, we defined significant peaks using ChIPOTle version 1.11 [66] with window size 300 bp, step size 38 bp. We selected the top 50 promoter peaks from each chromosome, based on p-value, for a total of 300 peaks, and analyzed the 1 kb sequence surrounding their centers with MEME [67]. We searched for 7–11 mer DNA motifs with parameters “-dna -mod zoops -minsites 20 -revcomp -minw 7 -maxw 11” and 5th markov model of all *C. elegans* promoter sequences as a background nucleotide distribution, and then searched for 12–18 mer DNA motifs with parameters “-dna -mod zoops -minsites 20 -revcomp -minw 12 -maxw 18” and the same background markov model. We confirmed that predicted motifs lie within ChIP peaks (Figure S2). We determined the genomic distributions of promoter-associated TF motifs by searching promoter regions (1 kb upstream from TSS) of all 20158 *C. elegans* genes (WS200) using MAST (Figure 5D) or FIMO (Figure S2) in the MEME suite [67]. Although the absolute values of motif occurrence varied depending on the p-value cutoff, the under-representation of Motifs 1 and 2 on the X chromosome was observed at multiple cutoffs. p-value cutoff used to search motifs in Figure 5D and Table S3: 10^−5^ (EFL-1, HLH-27), 10^−6^ (Motif 1, FLH-1), and 10^−7^ (NFI-1).

### GO Term Analysis

GO analysis was performed using GO-TermFinder [68], with p-value cut off of 0.01 (for LIN-54 bound genes) or 0.05 (for LIN-54 responsive genes) with Bonferroni correction for multiple hypothesis testing. The evidence code Inferred from Electronic Annotation (IEA) was excluded from the analysis.

### Microarray Analysis

Statistical analyses were performed using R, a system for statistical computation and graphics ([69]; http://www.r-project.org). The rma method in the affy package from Bioconductor was used in R to summarize probe level data and to normalize the dataset to remove across array variation [70], [71]. Log transformed data were used in subsequent analysis and plotting. WormBase version WS190 was used.To determine differentially expressed genes between wild-type and mutants, moderated T Statistics in limma [72] was used with p-value≤0.01, fold change ≥1.5. When multiple probes sets correspond to one gene, the average fold change was determined. Raw data from [25] was re-analyzed with the same criteria described above, and genes responsive to *efl-1(n3639)*, *dpl-1(n3316)*, *lin-35(n745)*, and *lin-54(n3423)* were clustered by the centroid-linkage hierarchical analysis (Cluster 3.0, [73]). Clusters were visualized with Java Treeview [74]. To calculate the percent of genes per chromosome responsive to DRM members, we used the number of genes common between the custom arrays of [25] and those represented on GeneChip *C. elegans* genome arrays (Affymetrix).

To estimate genes normally expressed in wild-type embryos or germlines, we utilized the detection (present/absent) call generated by the Affymetrix microarray suite. Each probe set received numeric score based on the detection calls (present = 1, marginal = 0, and absent = −1), and the sum of the score for three biological replicas were calculated for each probe set (i.e. present in all three replicas = 3). A gene was considered expressed if the average score was more than 1.5, and absent if less than −1.5. Our lists of expressed genes were comparable with those determined by SAGE analysis [38].

The microarray and ChIP data in this publication have been deposited in NCBI's Gene Expression Omnibus and are accessible through GEO Series accession number GSE28494. http://www.ncbi.nlm.nih.gov/geo/query/acc.cgi?acc=GSE28494

## Supporting Information

Figure S1 The conserved DRM complex, its binding to promoters of genes encoding DRM subunits, and disruption of its binding in the *lin-54(n2990)* mutant. *(A)* Cartoon represents the eight-subunit *C. elegans* DRM complex. Table shows DRM subunits and their homologs in the *D. melanogaster* dREAM/MMB complex and in the *H. sapiens* LINC/DREAM complex. *D. melanogaster* also has a paralogous tMAC complex that is testis-specific. A Myb subunit has not been identified in *C. elegans* DRM. *(B)* LIN-54 and other DREAM subunits bind to the 5′ ends (within 1 kb of TSS) of genes encoding DREAM subunits in worms (this study), flies [21], and humans [5], [7]. *LIN-54 binding at its own promoter is indicated here because a strong, broad, peak was observed. Because its mode is just inside the coding region it did not meet our definition of LIN-54 bound genes in Table S1. *(C)* DRM subunit binding in wild-type and *lin-54(n2990)* mutants, measured by ChIP-qPCR at the target promoters *lin-9* and *lin-54*. Binding is shown as the amount of DNA amplified in each ChIP sample relative to input, without setting the ratio in wild-type to 1.0 as in Figure 1C. Results from three independent experiments are shown. *(D)* Immunofluorescence of hermaphrodite germline nuclei with antibodies against DRM subunits LIN-54, DPL-1, LIN-9, or LIN-37 in wild-type, *lin-54(n2990)* and *lin-54(n3423)* at 20°C. Strength of chromosome-associated staining was scored blind and assigned a score of 3 (strong), 2 (moderate), 1 (weak), or 0 (none) from at least two independent experiments and at least 20 different germlines; average score shown. *lin-54(n3423)* null strain severely disrupts association of other DRM subunits and the *lin-54(n2990)* strain partially disrupts association. Nuclei scored in region from germline tip until mid-pachytene stage of meiosis, as indicated above. 1. Harrison et al. 2006 [4] 2. Korenjak et al. 2004 [2] 3. Lewis et al. 2004 (MMB also includes Rpd3 and L(3)MBT) [3] 4. Beall et al. 2007 (tMAC also includes Comr and Topi) [19] 5. Litovchick et al. 2007 [5] 6. Schmit et al. 2007 [7] 7. Detected only in MMB 8. Detected only in hDREAM 9. Detected only in LINC 10. Georlette et al. 2007 [21].
(TIF)

Figure S2 An additional motif enriched in LIN-54 bound promoters and location of Motif 1 relative to ChIP peak *(A)* Motif 2, enriched in LIN-54 bound promoters, and a related motif identified in *Drosophila* DREAM-bound promoters [21]. *(B)* Occurrence of Motif 2 in promoter regions of autosomal genes (gray bars) and X-linked genes (black bar). Motif 2 is under-represented within X-linked gene promoters (p-value<10^−5^). *(C)* The distance between the mode of LIN-54 ChIP peaks and the location of Motif 1. Based on criteria described in Materials and Methods, 356 genes contained both a LIN-54 ChIP-peak and Motif 1 within 1 kb upstream from their TSS. More than half of those promoters had ChIP-peak modes that lie within 100 bp from the putative E2F-LIN-54 binding consensus (Motif 1).
(TIF)

Figure S3 *lin-54(n2990)* mutants show similar, but weaker, phenotypes compared with *lin-54(n3423)* null mutants. *(A)* Wild-type (top) and *lin-54(n2290)* (bottom) young adult hermaphrodites stained for DNA. *lin-54* mutants exhibit an endomitotic oocyte (EMO) phenotype (left) which can result from various defects including defects in meiotic cell cycle, somatic sheath cell formation, or fertilization. *lin-54* mutants also exhibit inappropriately connected gut nuclei (right), which may result from defects in mitotic chromosome segregation. Table shows comparison of these phenotypes in *lin-54(n2990)* and *lin-54(n3423)* at 20°C and 25°C. M+Z− (homozygous animals from heterozygous mother); M−Z− (homozygous animals from M+Z− hermaphrodites). % EMO: the percentage of animals with EMO phenotype 24 hrs. after L4 stage. % gut bridges: percentage calculated as the number of gut nuclei with an obvious connection/total gut nuclei×100. *(B)* Dissected hermaphrodite germlines from wild-type (top), *lin-54(n2990)* (middle) and *lin-54(n3423)* (bottom) stained for DNA. Arrowheads indicate endomitotic oocytes. Box indicates region excised for germline microarray, chosen because germline nuclear morphology is similar between wild-type and mutant and because these stages precede re-activation of the X chromosome [26].
(TIF)

Figure S4 LIN-54, EFL-1, DPL-1, and LIN-35 co-regulated genes show chromosomal bias. Hierarchical clustering analysis of genes that changed expression in *efl-1(n3639)*, *dpl-1(n3316)*, *lin-35(n745)*, and/or *lin-54(n3423)* (left). The chromosomal distribution and the enriched Gene Ontology terms of ten clusters of genes are shown (right). p-value cutoff used for GO term search <0.01 with Bonferroni correction. NS = no significant GO found. * p-value<10^−5^.
(TIF)

Table S1 LIN-54 ChIP peak locations, bound genes, GO terms of bound genes, and genes commonly bound between *C. elegans* and *D. melanogaster* or human. *(Tab 1)* Genomic locations of LIN-54 ChIP peaks and overlapping or nearby genes. LIN-54 ChIP peaks from two biological replicas were analyzed and merged using the MA2C program. Peak modes that are intragenic, 5′ to gene, or 3′ to gene are indicated. *(Tab 2)* LIN-54 bound genes defined in this study. *(Tab 3)* Gene Ontology terms enriched in genes containing LIN-54 ChIP peaks within 1 kb from TSS (p-value<0.01) *(Tab 4)* Genes with worm-fly orthologs defined by HomoloGene that are commonly bound by LIN-54 in *C. elegans* and *D. melanogaster* [21]. *(Tab 5)* Genes with worm-human orthologs defined by HomoloGenes that are commonly bound by LIN-54 in *C. elegans* and human [5]. FDR = False Discovery Rate. For details see Materials and Methods.
(XLS)

Table S2 LIN-54 responsive genes and their GO terms. *(Tab 1)* Genes with changed expression in *lin-54(n2990)* embryos. *(Tab 2)* Genes with changed expression in *lin-54(n3423)* germlines. *(Tab 3)* Gene Ontology terms enriched in genes up-regulated or down-regulated in *lin-54(n2990)* embryos or *lin-54(n3423)* germline. (p-value<0.05). *(Tab 4)* Genes with both LIN-54 ChIP peaks in their promoters and changed expression in *lin-54* mutants (“bound and regulated”). *(Tab 5)* Enriched Gene Ontology terms of LIN-54 “bound and regulated” gene set. FDR = False Discovery Rate. logFC = log fold change. For details see Materials and Methods.
(XLS)

Table S3 Chromosomal distribution of *(A)* LIN-54 ChIP peaks, *(B)* Binding motifs for E2F-LIN-54 (Motif 1) and other transcription factors. p-value cutoff used to search motifs is 10^−5^ (EFL-1, HLH-27), 10^−6^ (Motif 1, FLH-1), and 10^−7^ (NFI-1). *(C)* LIN-54 responsive genes in embryos and germlines.
(PDF)
