# Supplementary figures and images for: Chromosome-Biased Binding and Gene Regulation by the Caenorhabditis elegans DRM Complex
Source: PLoS Genet. 2011 May 12;7(5):e1002074. doi: 10.1371/journal.pgen.1002074 (PMC3093354; doi:10.1371/journal.pgen.1002074)

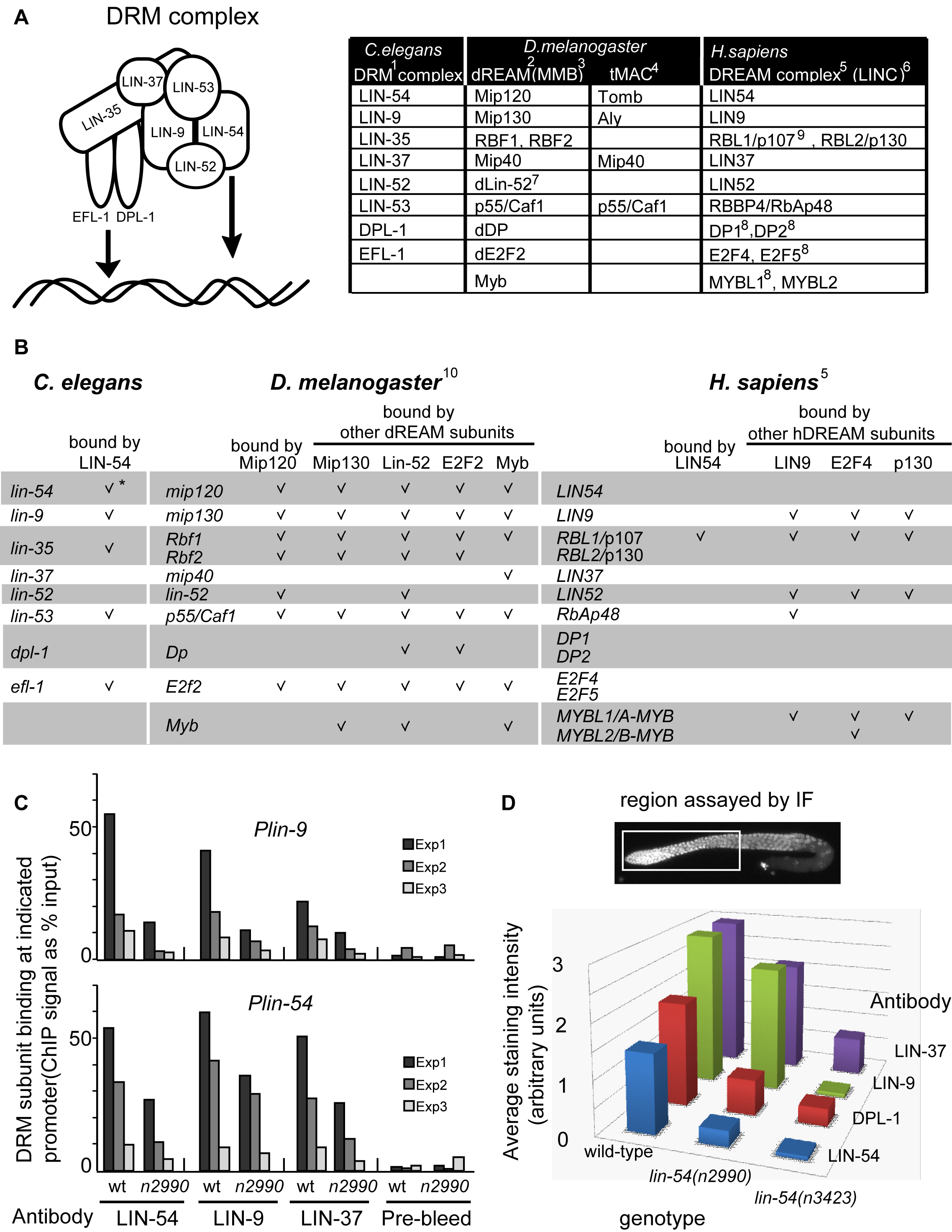

Supplement: Figure S1 — The conserved DRM complex, its binding to promoters of genes encoding DRM subunits, and disruption of its binding in the lin-54(n2990) mutant. (A) Cartoon represents the eight-subunit C. elegans DRM complex. Table shows DRM subunits and their homologs in the D. melanogaster dREAM/MMB complex and in the H. sapiens LINC/DREAM complex. D. melanogaster also has a paralogous tMAC complex that is testis-specific. A Myb subunit has not been identified in C. elegans DRM. (B) LIN-54 and other DREAM subunits bind to the 5′ ends (within 1 kb of TSS) of genes encoding DREAM subunits in worms (this study), flies [21], and humans [5], [7]. *LIN-54 binding at its own promoter is indicated here because a strong, broad, peak was observed. Because its mode is just inside the coding region it did not meet our definition of LIN-54 bound genes in Table S1. (C) DRM subunit binding in wild-type and lin-54(n2990) mutants, measured by ChIP-qPCR at the target promoters lin-9 and lin-54. Binding is shown as the amount of DNA amplified in each ChIP sample relative to input, without setting the ratio in wild-type to 1.0 as in Figure 1C. Results from three independent experiments are shown. (D) Immunofluorescence of hermaphrodite germline nuclei with antibodies against DRM subunits LIN-54, DPL-1, LIN-9, or LIN-37 in wild-type, lin-54(n2990) and lin-54(n3423) at 20°C. Strength of chromosome-associated staining was scored blind and assigned a score of 3 (strong), 2 (moderate), 1 (weak), or 0 (none) from at least two independent experiments and at least 20 different germlines; average score shown. lin-54(n3423) null strain severely disrupts association of other DRM subunits and the lin-54(n2990) strain partially disrupts association. Nuclei scored in region from germline tip until mid-pachytene stage of meiosis, as indicated above. 1. Harrison et al. 2006 [4] 2. Korenjak et al. 2004 [2] 3. Lewis et al. 2004 (MMB also includes Rpd3 and L(3)MBT) [3] 4. Beall et al. 2007 (tMAC also includes Comr and T [file pgen.1002074.s001.tif]

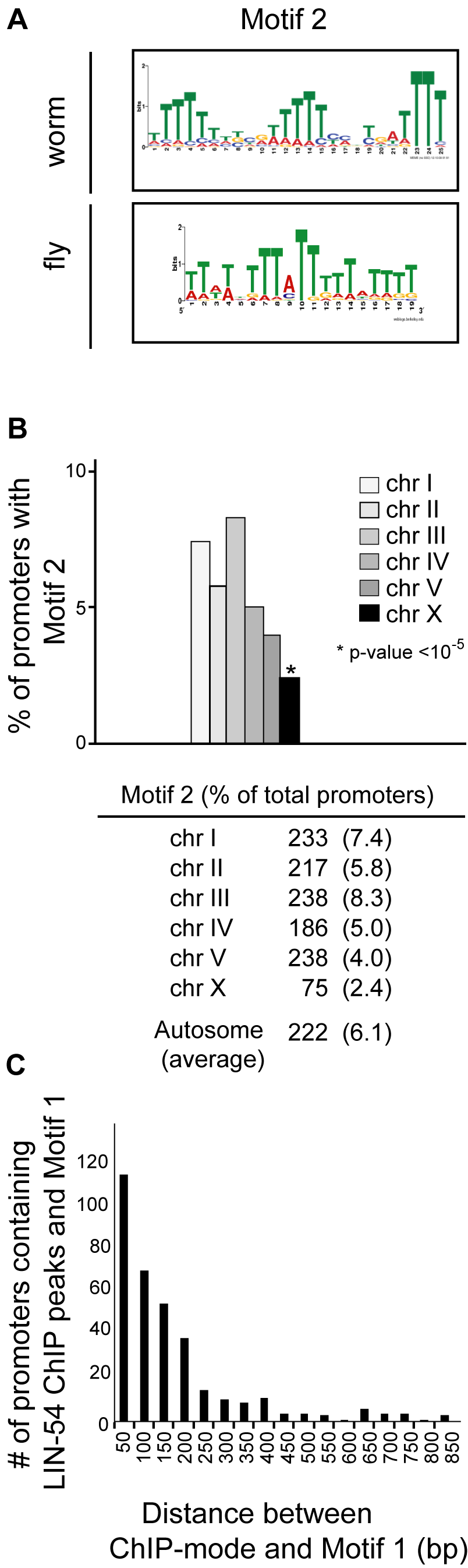

Supplement: Figure S2 — An additional motif enriched in LIN-54 bound promoters and location of Motif 1 relative to ChIP peak (A) Motif 2, enriched in LIN-54 bound promoters, and a related motif identified in Drosophila DREAM-bound promoters [21]. (B) Occurrence of Motif 2 in promoter regions of autosomal genes (gray bars) and X-linked genes (black bar). Motif 2 is under-represented within X-linked gene promoters (p-value<10−5). (C) The distance between the mode of LIN-54 ChIP peaks and the location of Motif 1. Based on criteria described in Materials and Methods, 356 genes contained both a LIN-54 ChIP-peak and Motif 1 within 1 kb upstream from their TSS. More than half of those promoters had ChIP-peak modes that lie within 100 bp from the putative E2F-LIN-54 binding consensus (Motif 1). (TIF) [file pgen.1002074.s002.tif]

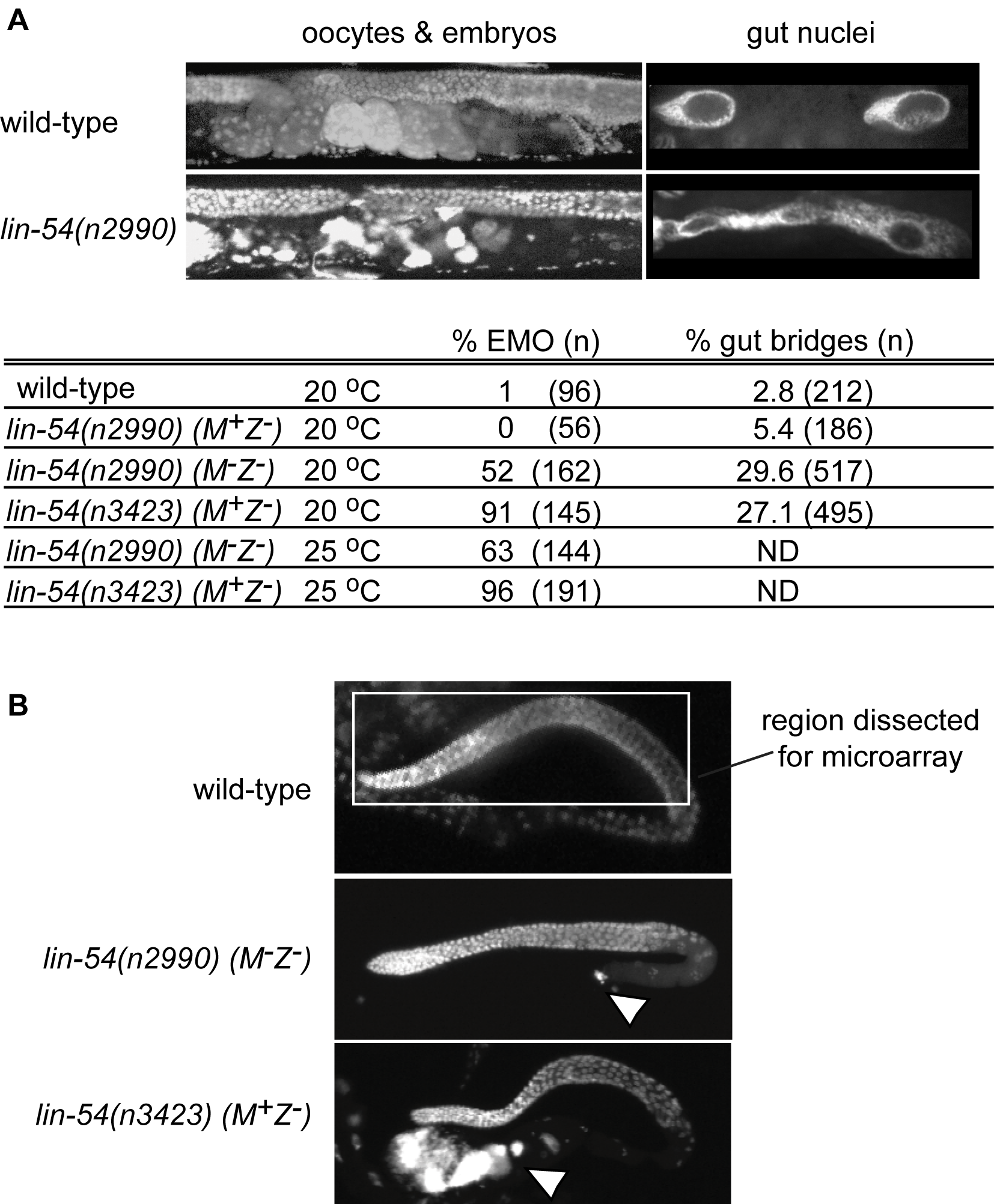

Supplement: Figure S3 — lin-54(n2990) mutants show similar, but weaker, phenotypes compared with lin-54(n3423) null mutants. (A) Wild-type (top) and lin-54(n2290) (bottom) young adult hermaphrodites stained for DNA. lin-54 mutants exhibit an endomitotic oocyte (EMO) phenotype (left) which can result from various defects including defects in meiotic cell cycle, somatic sheath cell formation, or fertilization. lin-54 mutants also exhibit inappropriately connected gut nuclei (right), which may result from defects in mitotic chromosome segregation. Table shows comparison of these phenotypes in lin-54(n2990) and lin-54(n3423) at 20°C and 25°C. M+Z− (homozygous animals from heterozygous mother); M−Z− (homozygous animals from M+Z− hermaphrodites). % EMO: the percentage of animals with EMO phenotype 24 hrs. after L4 stage. % gut bridges: percentage calculated as the number of gut nuclei with an obvious connection/total gut nuclei×100. (B) Dissected hermaphrodite germlines from wild-type (top), lin-54(n2990) (middle) and lin-54(n3423) (bottom) stained for DNA. Arrowheads indicate endomitotic oocytes. Box indicates region excised for germline microarray, chosen because germline nuclear morphology is similar between wild-type and mutant and because these stages precede re-activation of the X chromosome [26]. (TIF) [file pgen.1002074.s003.tif]

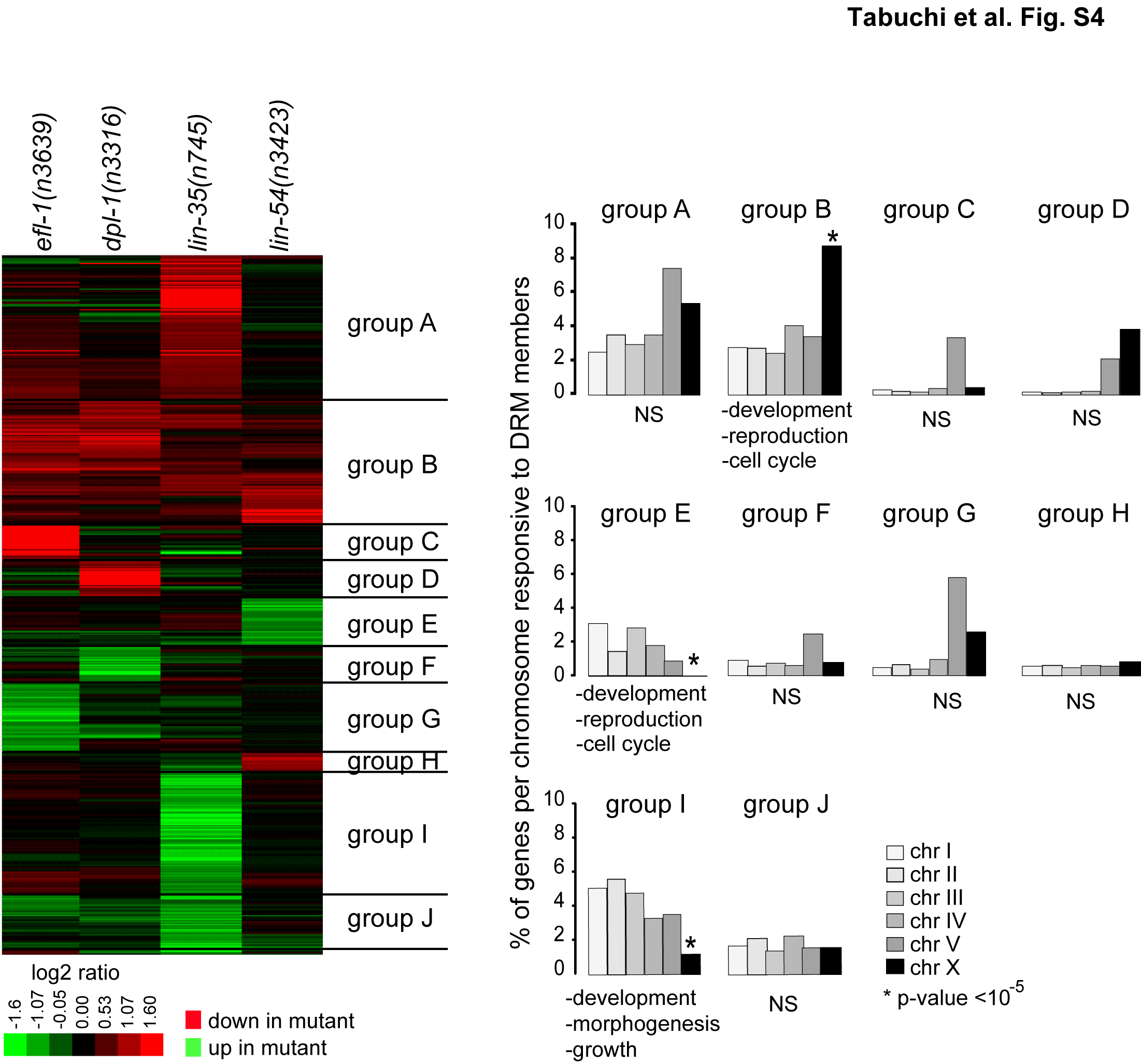

Supplement: Figure S4 — LIN-54, EFL-1, DPL-1, and LIN-35 co-regulated genes show chromosomal bias. Hierarchical clustering analysis of genes that changed expression in efl-1(n3639), dpl-1(n3316), lin-35(n745), and/or lin-54(n3423) (left). The chromosomal distribution and the enriched Gene Ontology terms of ten clusters of genes are shown (right). p-value cutoff used for GO term search <0.01 with Bonferroni correction. NS = no significant GO found. * p-value<10−5. (TIF) [file pgen.1002074.s004.tif]
