# Supplementary material for: Chromosome-Biased Binding and Gene Regulation by the Caenorhabditis elegans DRM Complex
Source: PLoS Genet. 2011 May 12;7(5):e1002074. doi: 10.1371/journal.pgen.1002074 (PMC3093354; doi:10.1371/journal.pgen.1002074)
Supplement: Table S3 — Chromosomal distribution of (A) LIN-54 ChIP peaks, (B) Binding motifs for E2F-LIN-54 (Motif 1) and other transcription factors. p-value cutoff used to search motifs is 10−5 (EFL-1, HLH-27), 10−6 (Motif 1, FLH-1), and 10−7 (NFI-1). (C) LIN-54 responsive genes in embryos and germlines. (PDF) [file pgen.1002074.s007.pdf]

LIN-54 binding and Motif 1 are under-represented on the X chromosome

A Distribution of LIN-54 ChIP-peaks across the genome

	LIN-54 ChIP peaks (per Mb)	Promoter-associated LIN-54 ChIP peaks (%)
chr I	400 (27)	322 (10)
chr II	291 (19)	262 (7)
chr III	429 (31)	372 (13)
chr IV	325 (19)	264 (7)
chr V	402 (19)	288 (5)
chr X	145 (8)	64 (2)
Autosome (average)	369 (23)	302 (8)

B Distribution of gene promoters containing various motifs across the genome

	Motif 1 (%)	EFL-1 (%)	HLH-27 (%)	FLH-1 (%)	NFL-1 (%)
chr I	105 (3.9)	226 (8.3)	90 (3.3)	29 (1.1)	28 (1.0)
chr II	90 (2.7)	179 (5.4)	87 (2.6)	27 (0.8)	54 (1.6)
chr III	91 (3.7)	179 (7.2)	83 (3.3)	32 (1.3)	35 (1.4)
chr IV	82 (2.6)	163 (5.2)	101 (3.2)	33 (1.1)	28 (0.9)
chr V	125 (2.6)	261 (5.3)	149 (3.1)	51 (1.0)	64 (1.3)
chr X	17 (0.6)	166 (6.2)	63 (2.4)	23 (0.9)	20 (0.7)
Autosome (average)	99 (3.0)	202 (6.1)	102 (3.1)	34 (1.0)	42 (1.3)

C Distribution of LIN-54 responsive genes across the genome

	lin-54(n2990) embryo		*lin-54(n3423)* germline	
	up (%)	down (%)	up (%)	down (%)
chr I	128 (5.0)	35 (1.4)	19 (0.74)	28 (1.1)
chr II	152 (4.9)	57 (1.8)	14 (0.45)	41 (1.3)
chr III	135 (5.8)	41 (1.8)	17 (0.73)	27 (1.2)
chr IV	108 (3.9)	50 (1.8)	11 (0.39)	51 (1.8)
chr V	110 (2.5)	61 (1.4)	15 (0.34)	40 (0.9)
chr X	45 (1.8)	55 (2.2)	1 (0.04)	64 (2.6)
Autosome (average)	127(4.2)	49 (1.6)	15 (0.50)	37 (1.2)
